# Supplementary material for: Identification of photoperiod-induced specific miRNAs in the adrenal glands of Sunite sheep (Ovis aries)
Source: Front Vet Sci. 2022 Jul 22;9:888207. doi: 10.3389/fvets.2022.888207 (PMC9354845; doi:10.3389/fvets.2022.888207)
Supplement: Supplementary file 1 [file Data_Sheet_1.ZIP › Supplementary file/miRDeep2 file.zip!/miRDeep2 file/Know_Structure/SPLP42A3/oar-mir-10b.pdf]

miRBase precursor	: oar-mir-10b
Total read count	: 541066
oar-miR-10b read count	: 538785
remaining reads	: 2281

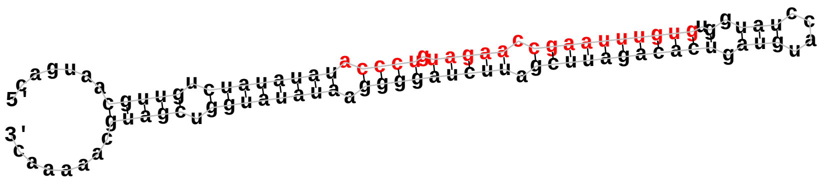

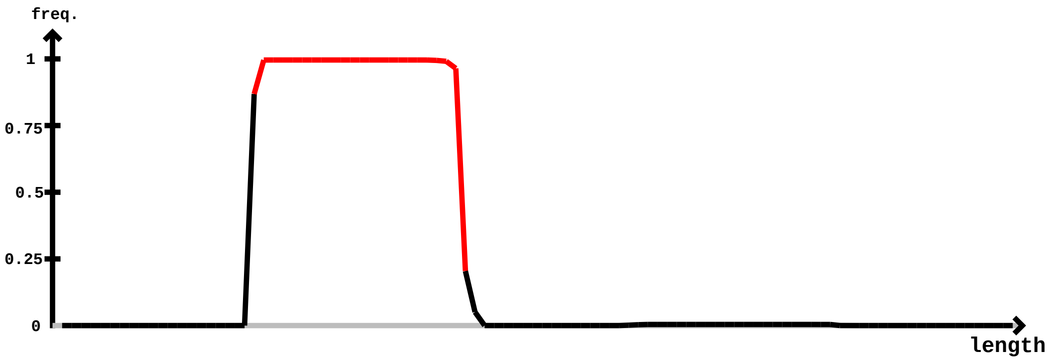

oar-miR-10b

```
5'- caguaacguugucuauauauacccuguagaaccgaauuugugugguauccauguagucacagauucgauucuaggggaauauauggucgaugcaaaaac   -3'    exp
    ......(((((.((((((((.((((.(((((.((((((((((.(.(((....))).))))))))))).))))))))).)))))))).))))).......    reads      mm         sample
    ..................auacccuguagaaccgaauuug...........................................................    1          0          seq
    ..................auacccuguagaaccgaauuugu..........................................................    10         0          seq
    ...................uacccuguagaaccgaau..............................................................    34         0          seq
    ...................uacccuguagaaccgaauu.............................................................    725        0          seq
    ...................uacccuguagaaccgaauuu............................................................    1605       0          seq
    ...................uacccuguagaaccgaauuug...........................................................    12634      0          seq
    ...................uacccuguagaaccgaauuugu..........................................................    382583     0          seq
    ...................uacccuguagaaccgaauuugug.........................................................    61573      0          seq
    ...................uacccuguagaaccgaauuugugu........................................................    10673      0          seq
    ...................uacccuguagaaccgaauuugugug.......................................................    6          0          seq
    ....................acccuguagaaccgaau..............................................................    1          0          seq
    ....................acccuguagaaccgaauu.............................................................    52         0          seq
    ....................acccuguagaaccgaauuu............................................................    226        0          seq
    ....................acccuguagaaccgaauuug...........................................................    1716       0          seq
    ....................acccuguagaaccgaauuugu..........................................................    28616      0          seq
    ....................acccuguagaaccgaauuugug.........................................................    21592      0          seq
    ....................acccuguagaaccgaauuugugu........................................................    16637      0          seq
    ....................acccuguagaaccgaauuugugug.......................................................    43         0          seq
    ....................acccuguagaaccgaauuugugugg......................................................    3          0          seq
    .....................cccuguagaaccgaauuug...........................................................    1          0          seq
    .....................cccuguagaaccgaauuugu..........................................................    16         0          seq
    .....................cccuguagaaccgaauuugug.........................................................    8          0          seq
    .....................cccuguagaaccgaauuugugu........................................................    17         0          seq
    ......................ccuguagaaccgaauuugu..........................................................    5          0          seq
    ......................ccuguagaaccgaauuugug.........................................................    2          0          seq
    ......................ccuguagaaccgaauuugugu........................................................    2          0          seq
    .......................cuguagaaccgaauuugu..........................................................    1          0          seq
    .......................cuguagaaccgaauuugugu........................................................    3          0          seq
    .........................................................cacagauucgauucuagggga.....................    1          0          seq
    .........................................................cacagauucgauucuaggggaa....................    2          0          seq
    .........................................................cacagauucgauucuaggggaau...................    8          0          seq
    ..........................................................acagauucgauucuagggga.....................    17         0          seq
    ..........................................................acagauucgauucuaggggaa....................    24         0          seq
    ..........................................................acagauucgauucuaggggaau...................    731        0          seq
    ..........................................................acagauucgauucuaggggaaua.................    4          0          seq
```

oar-miR-10b

```
caguaacguugucuauauauacccuguagaaccgaauuugugugguauccauguagucacagauucgauucuaggggaauauauggucgaugcaaaaac
...........................................................cagauucgauucuagggg......................    1     0     seq
...........................................................cagauucgauucuagggga.....................    16    0     seq
...........................................................cagauucgauucuaggggaa....................    16    0     seq
...........................................................cagauucgauucuaggggaau...................    866   0     seq
...........................................................cagauucgauucuaggggaaua..................    21    0     seq
...........................................................cagauucgauucuaggggaauau.................    5     0     seq
............................................................agauucgauucuagggga.....................    4     0     seq
............................................................agauucgauucuaggggaa....................    5     0     seq
............................................................agauucgauucuaggggaau...................    490   0     seq
............................................................agauucgauucuaggggaaua..................    46    0     seq
............................................................agauucgauucuaggggaauau.................    23    0     seq
.............................................................gauucgauucuaggggaauau.................    1     0     seq
```
